# A Kirigami Approach to Forming a Synthetic Buckliball

**DOI:** 10.1038/srep33016

**Published:** 2016-09-09

**Authors:** Sen Lin, Yi Min Xie, Qing Li, Xiaodong Huang, Shiwei Zhou

**Affiliations:** 1 Centre for Innovative Structures and Materials, School of Engineering, RMIT University, GPO Box 2476, Melbourne 3001, Australia; 2 School of Aerospace, Mechanical and Mechatronic Engineering, The University of Sydney, NSW 2006, Australia

## Abstract

The shape transformation of some biological systems inspires scientists to create sophisticated structures at the nano- and macro- scales. However, to be useful in engineering, the mechanics of governing such a spontaneous, parallel and large deformation must be well understood. In this study, a kirigami approach is used to fold a bilayer planar sheet featuring a specific pattern into a buckliball under a certain thermal stimulus. Importantly, this prescribed spherical object can retract into a much smaller sphere due to constructive buckling caused by radially inward displacement. By minimizing the potential strain energy, we obtain a critical temperature, below which the patterned sheet exhibits identical principal curvatures everywhere in the self-folding procedure and above which buckling occurs. The applicability of the theoretical analysis to the self-folding of sheets with a diversity of patterns is verified by the finite element method.

Currently, the inability to rapidly and precisely fabricate highly complex sophisticated structures at the micro- and nano-scales has considerably impeded their applications^1^. Recently, an alternative approach has been developed via the folding planar sheets (panels) with prescribed features, such as ridges and grooves, into sophisticated 3D objects^2^. Because these sheets can be quickly fabricated with unprecedented precision using lithographic techniques^3^ and the self-folding procedure can spontaneously occur in a parallel manner, this approach is particularly attractive for assembling complex structures efficiently and economically, and it has the potential to overcome fabrication difficulties at small scales^4^.

The technique of fabricating 3D objects from planar sheets is referred to as the origami approach because the original idea originates from origami, the traditional art of paper-folding, which is popular in China and Japan. The origami approach has been developed to produce fairly complex structures, such as a flapping bird by folding a sandwich-like sheet along predesigned veins^5^, and a periodic structure consisting of extruded cubes, which can be actively deformed into numerous specific shapes through embedded actuation^6^. Additionally, novel techniques, such as direct ink writing, exhibit attractive features to realize the design rules that are required for an origami-based structure^7^. Instead of folding a flawless sheet along some specified creases as in the origami approach, the kirigami method signifies an alternative approach to forming 3D structures from a patterned planar sheet, which relies on the folding of a sheet that is perforated into some specific patterns. The presence of apertures allows more flexible control of the self-folding process, therefore enabling programmable fabrication of complex structures.

The kirigami approach is demonstrated in nature in the formation of the curled leaves of some tropical plants^8^. This approach was recently exploited to fabricate biomimetic robotics by utilizing the active response of electroactive polymers and shape memory polymers^9^. Buckling-induced deformation was used in the kirigami approach to fabricate diverse 3D membrane architectures by releasing a pre-strained stretchable substrate on which a thin layer of metal was cut into predefined patterns^10^. More recently, researchers have identified multilevel hierarchical patterns as a way to transform the sheet into many desirable programmed shapes^11^,^12^. In the kirigami approach, the diversity of the cutting patterns greatly expands the design space, and it is, therefore, worthy of further investigation. This approach is expected to be capable of fabricating synthetic materials or devices for a range of novel applications, such as drug delivery agents^13^, soft robotics^14^, optical metamaterials^15^, solar cells^16^ and untethered microsurgical tools^17^, which will potentially lead to immense scientific and economic benefits.

This study aims to employ the kirigami approach to producing a buckliball, a collapsible structure notable for its opening and closing behaviors, similar to Hoberman Toy Spheres^18^ and the morphing of some viruses. With 24 circular dimples on a spherical shell, a buckliball can easily shrink into an enclosed ball-like structure that occupies nearly half of its original space. This special geometry enables us to achieve a much more sophisticated configuration than previous studies^9^,^10^,^11^,^12^,^19^. This paper focuses on the response of patterned planar sheet to a thermal stimulus, which is more applicable to bionics and medical science than electrical and mechanical stimuli. Specifically, the sheet will undergo the following two major deformation steps: (1) It bends homogenously, during which the principal curvatures at any point are predominantly equal in all directions. Different from most of the existing self-folding structures, buckling is strictly prohibited in this step; otherwise, the shell will not bend into a spherical shape but rather will present saddle-like shapes and curled edges. (2) Buckling-induced deformation becomes dominant in the subsequent retraction of the buckliball that is formed in the previous step. Buckling is one of the most important factors in the kirigami approach and subsequent morphing process. Yet, precise buckling control remains challenging for the kirigami approach, primarily due to the inherent coupling between the different components.

The planar sheet consists of two polymer layers, and such a soft material can rapidly respond to thermal stimuli and bear external osmotic pressure. The material distribution in the 2D design domain of a planar sheet determines its out-of-plane deformation and should be purposely patterned to achieve the desired final shape. To obtain such a perquisite kirigami pattern, the buckliball is first divided into several equal parts before being flattened into a plane. By minimizing the potential strain energy over the sheet, the relationship between the principal curvatures and temperature can be established and computationally verified. The creation of a buckliball by folding a patterned sheet and its subsequent buckling-induced retraction is numerically simulated using finite element analysis in the commercial FE software Abaqus. The influence of the sheet pattern on the folding procedure is investigated by testing the configurational diversity, including hole-less petals and petals dotted with circular apertures.

## Methods

### Strategic Pattern Design

**Figure 1:**
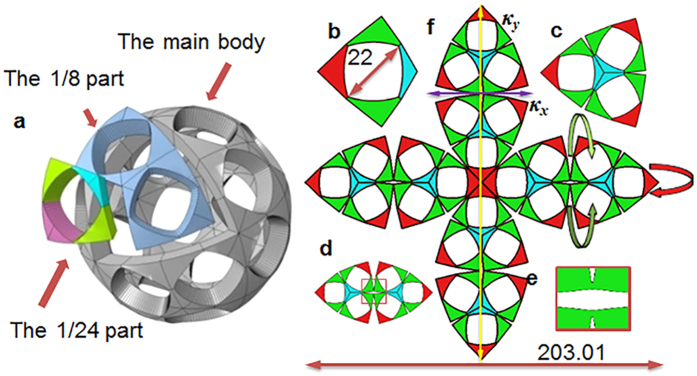
(**a**) The partition of a buckliball with rounded-square apertures into 1/8 and 1/24 parts; (**b**) 1/24 of a buckliball; (**c**) 1/8 of a buckliball; and (**d**) 1/4 of the pattern. (**e**) The magnified joint in (**d**). (**f**) The pattern is divided into red, green and cyan regions in accordance with bending performance.

Our analysis can be used for buckliballs of any thickness and arbitrary geometry. To meet the requirements of macro/micro applications, we choose a structure where the size of the target buckliball (Fig. 1a) is dimensionless, and its external and internal radii are 33 and 31 units, respectively. The aperture has a rounded-square shape in which the distance between the opposite vertices is 22. To obtain a buckliball with a large volume retraction ratio, a 2D pattern is first designed using a superformula, which can generate a variety of complex shapes^20^. Compared with circular apertures, the volume retraction ratio of a buckliball with rounded-square apertures can be improved by 8.65%^20^. Because the shape transformation is inherently reversible, an intuitive way to obtain the planar pattern of a 3D object is by unfolding it^21^. Recently, an algorithm was developed to design the folding creases for the art of origami^22^,^23^. Unfortunately, this algorithm is inapplicable to the design of the kirigami pattern. To address this issue, the unfolding procedure of the model in Fig. 1a is split into the following two steps: partition and disassembling. As a buckliball dotted by evenly distributed apertures is geometrically analogous to a tetrahexahedron with a hole on each face, it is straightforward to split it into 24 identical parts, as shown in Fig. 1a. The base cell, or a 1/24 part of the buckliball, is composed of one aperture and four curved triangle parts (denoted by two similar green triangles, one pink triangle, and one cyan triangle).

Folding 2D nets to 3D polyhedrons along the shortest paths (geodesics) in the configuration space is one of the two geometric principles of the design of self-foldable structures^24^. While this principle is effective for thermally responsive sheets whose bending is restricted to the hinge-like creases in the origami approach^25^, it is unsuitable for the bending resulting from material swelling/shrinking over an entire domain with the kirigami procedure. To obtain a strategic pattern so that the soft material can bend in the same way as a squid stretches its tentacles, the base cell should be first squeezed into a sheet by strengthening the arc length of all edges, forming a flat polyhedral face as shown in Fig. 1b.

It is helpful to make the 2D pattern doubly symmetric because this will lead to symmetric folding which is beneficial to effectively reduce potential mismatches^26^. Therefore, the three pieces of a polyhedral face shown in Fig. 1b are first united by sharing the edges of the cyan parts, constructing a part featuring 3-fold rotational symmetry (Fig. 1c). To achieve bilateral symmetry, the two parts shown in Fig. 1c are further connected at the vertexes of the green triangles (Fig. 1d). Because such vertexes are liable to induce numerical singularity in a finite element analysis, these single-nodal joints are artificially eliminated by filling materials into their adjacent areas, which is magnified in Fig. 1e. By rotating the pattern in Fig. 1d three times by 90°, 180° and 270° about its leftmost vertex, three similar petals are obtained. The combination of these petals, therefore, forms a square symmetric pattern featuring a span of 203.01 in the horizontal and vertical directions (Fig. 1f).

## Results

### Theory of Curvature Variation

**Figure 2:**
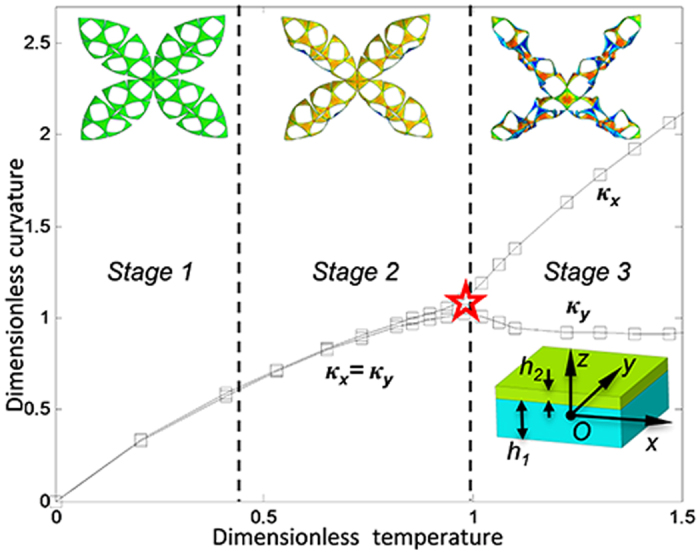
The relationship between the principal curvatures and temperature. In Stages 1 and 2, *κ*_*x*_ = *κ*_*y*._ Beyond the critical temperature, marked by a red star, the longitudinal and transverse principal curvatures deviate in Stage 3. The three top insets illustrate the typical bending states; the lower-right inset shows the thickness of the layers and the coordinate system.

In addition to the geometric pattern, the self-folding process depends on other factors, such as material properties, type of stimulus and layer thickness. For simplicity, this study is limited to a bilayer polymer sheet whose bending is attributed to the disparity of the thermal expansion coefficients in the top and bottom layers. To illustrate the nature of the bending, we consider a shell with a total thickness of *h* = *h*_1_ + *h*_2_. The bottom active layer and the top passive layer are tightly bonded, and their thicknesses are *h*_1_ and *h*_2_, respectively (shown in the lower-right inset of Fig. 2). The thickness ratio of the bottom layer to top layer is fixed as *h*_1_:*h*_2_ = 3:1. To obtain a spherical shell as shown in Fig. 1a, the longitudinal principal curvature *κ*_*y*_ and the transverse principal curvature *κ*_*x*_ are used to depict the shape of the bilayer sheet undergoing deformation. The direction of these two curvatures is illustrated in Fig. 1. To yield a buckliball, *κ*_*x*_ = *κ*_*y*_ must be maintained in the self-folding process. Upon determination of the pattern, the remaining geometric parameter that could affect the self-folding is the layer thickness.

Prior to the theoretical analysis, numerical simulations are conducted to investigate the role of thickness in the self-folding process. The simulations reveal that there are three major stages in the folding of the patterned sheet, as illustrated in Fig. 1f if its total thickness is as small as *h* = 0.2. Because the ratio of the shell thickness to its out-of-plane deformation is relatively small, the folding is subject to pure linear deformation, which is governed by the classic thin-shell theory in Stage 1. In Stage 2, the transverse displacements and rotations become relatively large compared with the vertical displacement; thus, a nonlinear deformation is dominant, and the increase of the principal curvatures becomes nonlinear with respect to the temperature increase. Nevertheless, the values of longitudinal principal curvature *κ*_*y*_ and the transverse principal curvatures *κ*_*x*_ remain the same in Stage 2 as in Stage 1. As a result, the shell can form a spherical surface in the first two stages, as shown in the upper-left and upper-middle insets in Fig. 2. However, the spherical surface will become a saddle shape, and the petal edges are curled in Stage 3 (shown in the upper-right inset in Fig. 2) when the temperature reaches a critical point, denoted by the red star in Fig. 2. In Stage 3, the petals bend more profoundly along the transverse direction than the longitudinal direction, resulting in curled edges that widely exist in nature. For instance, many leaves present such a shape when exposed to a dry climate for a long time^27^. Unlike our approach, in which the thermal stimulus serves as the driving force, the evaporation of water is the reason of the curled leaves. However, both the thermal stimulus and the evaporation of water allow the potential strain energy to approach the minimal point^28^.

The previous theoretical analysis of such a folding procedure was mainly limited to material properties, such as Poisson’s ratio, Young’s modulus, the thermal expansion coefficient,^19^ and some simple geometrical parameters, e.g., layer thickness^29^. However, the sheet pattern, to the best of our knowledge, has not been previously explored. As the primary premise of the kirigami approach is attributed to the diversity of shell patterns, it is necessary to theoretically analyze this approach, which is analogous to many naturally occurring shape transformations.

**Figure 3:**
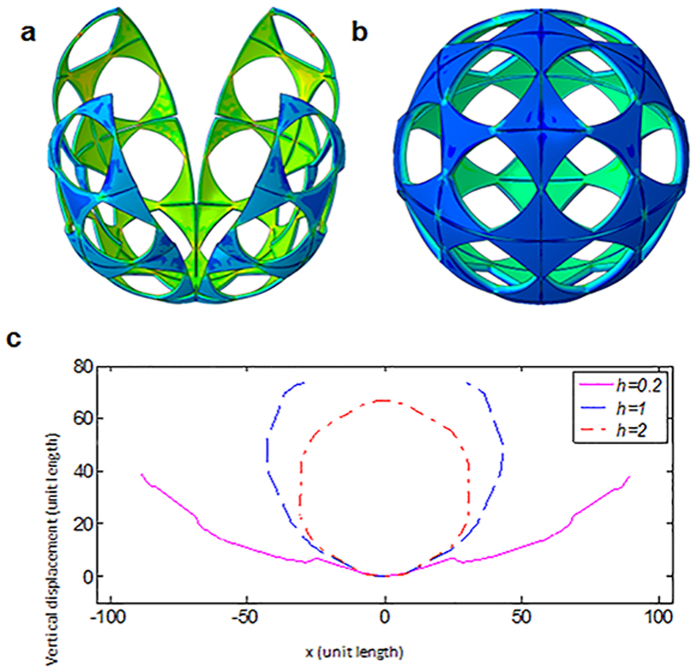
The perspective views of a deformed sheet with a similar pattern but different thicknesses of (**a**) *h* = 1 and (**b**) *h* = 2; (**c**) the cross-sectional profiles of the deformed sheet with *h* = 0.2, *h* = 1 and *h* = 2.

To avoid unexpected folding patterns, such as the saddle-like shapes and curled edges observed in Fig. 2, we investigate the association of the bending mode with the thickness, thereby determining the two distinct bending modes, as shown in Fig. 3. When *h* = 1, the shell folds into an ellipse-like object (the upper-left picture in Fig. 3), but the four petals cannot be merged due to the geometric constraint of these four petals being originally specified for a sphere. Continuing to increase the total thickness to *h* = 2, a buckliball gradually takes the shape shown in the upper-right inset in Fig. 3. The bottom graphical inset in Fig. 3 illustrates the cross-sectional profile (*x*-*z* plane) of the folded shell in the abovementioned cases. When the thickness is small (*h* = 0.2), the cross-sectional profile (solid pink curve) is approximately a part of a circle with a large radius. However, its buckle-induced deformation at the petal edges is not reflected. With an increase in the thickness, the cross section bends more severely and finally converges to a circle (the red dotted shape in Fig. 3).

The above simulations schematically reveal the dependence of the self-folding modes on the shell thickness. Hereafter, several simulations using different thicknesses clearly indicate that a critical temperature exists in the folding process, beyond which *κ*_*x*_ and *κ*_*y*_ bifurcate. When the temperature is confined to a limited range, self-folding of this planar sheet can be controlled by changing its thickness. Correspondingly, a critical thickness similar to the critical temperature can be determined. To obtain the critical value, we minimize the potential energy of the sheet perforated into a specified pattern.

At any point, three displacement components (*u*_1_, *u*_2_ and *u*_3_ in the *x*, *y* and *z*-axes, respectively) in the neutral plane of the plate are assumed as^30^:


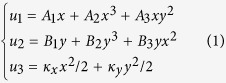


where *x*, *y* and *z* are the coordinates of this point in a Cartesian coordinate system, and *A*_*i*_ and *B*_*i*_ are the parameters to be determined. This assumption is based on the classic Kirchhoff plate theory^28^ (the derivation is provided in the Supplementary Information (SI)). The total strain *ε*_*αβ*_ (*α*, *β* = 1 or 2) can be formulated as:


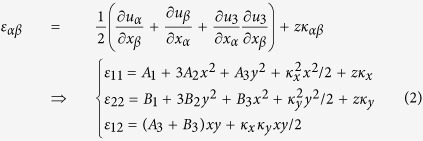


The stain energy density per thickness *U*_*ti*_ (*i* = 1, 2 denotes the layer sheet) can be represented as:


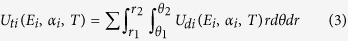


where *E*_*i*_ and *α*_*i*_ denote the Young’s modulus and the thermal expansion coefficient of each layer, respectively, and *U*_*di*_ is the strain energy density. According to Eq. (S1), the strain energy density *U*_*di*_ depends on stress *σ*, which is a function of temperature *T,* as expressed in Eq. (S2). Note that in Eq. (3), the Cartesian coordinate system has been converted to a polar coordinate system through *x* = *r* cos *θ* and *y* = *r* sin *θ*. The aperture shape is determined by the Gielis’ superformula^31^ as:


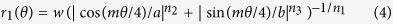


where the parameters are given as *a* = *b* = 0, *m* = 4, *n*_1_ = 150, *n*_2_ = *n*_3_ = 100, and *w* = 18. Considering the sophistication of using Eq. (4) in subsequent derivations, the aperture edge is divided into 4 elliptical arcs; each of these arcs can be further simplified (as shown in the *SI*). Due to the thickness being small relative to the other dimensions, the sheet can be approximated as a plate. Therefore, the strain energy *U* can be obtained by integrating the strain energy density *U*_*ti*_ in the *z* direction given as:


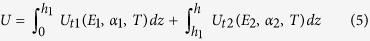


The strain energy in Eq. (5) relies on *U*_*ti*_ in Eq. (3). For instance, the strain energy is the energy of the base cell if the domain is confined to the shape shown in Fig. 1b. However, the strain energy of the petal can be determined using coordinate transformation in the integration, as each petal comprises six identical base cells. The detailed derivation is provided in the *SI*. Because the partial derivative of the total energy with respect to the unknown parameters *A*_*i*_ and *B*_i_ should be equal to zero, i.e., ∂*U*/∂*A*_*i*_ = 0, ∂*U*/∂*B*_*i*_ = 0, *A*_*i,*_ *B*_*i*_ can be determined. As a result, the partial derivatives of the total energy with respect to the two principal curvatures can also be obtained. A general expression of the total strain energy *U* for complex structures is provided in the Supplementary Information. Using our choice of buckliball geometry and the parameters *h*_1_ = 0.75 h, Poisson’s ratio *μ* = 0.3, *α*_1_ = 0, *α*_2_ = −0.005/°C, *E*_1_ = 3 MPa and *E*_2_ = 0.8 MPa (see the description in the next section) together with ∂*U*/∂*κ*_*x*_ = 0 and ∂*U*/∂*κ*_*y*_ = 0, we obtain:


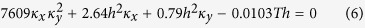



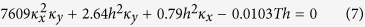


With the elimination of temperature *T* in Eqs (6) and (7), a simplified expression can be obtained as:


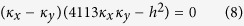


Two solutions of this equation, specifically *κ*_*x*_ = *κ*_*y*_ and 4113*κ*_*x*_*κ*_*y*_ = *h*^2^, characterize two explicit equilibrium states. The first signifies that the two principal curvatures must be strictly the same, as demonstrated in Stage 1 and Stage 2 shown in Fig. 2. The second solution implies that the product of the two principal curvatures is a constant in terms of thickness. As shown in Fig. 2, the two principal curvatures deviate in Stage 3, and the bifurcation point must satisfy both of these two solutions. Therefore, a critical curvature can be obtained as:


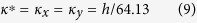


Eq. (9) indicates that the critical curvature is linear with respect to the sheet thickness. Substituting the critical curvature into Eq. (6), a critical temperature is thus determined as:


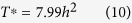


The expression of the critical temperature in Eq. (10) indicates that the thinner the sheet, the lower the critical temperature. For the final temperature *T* = 45.2 °C, which was determined in the previous simulation tests, the critical thickness is h^*^ = 1.78. Therefore, any patterned sheet that is thinner than this critical value is unable to self-fold into a perfect spherical configuration because these two principal curvatures would deviate in Stage 3 (i.e., *κ*_*x*_ > *κ*_*y*_). In contrast, for a sheet thicker than this critical value, the folding is limited in Stages 1 and 2 (*κ*_*x*_ = *κ*_*y*_), and a buckliball can be formed.

**Figure 4:**
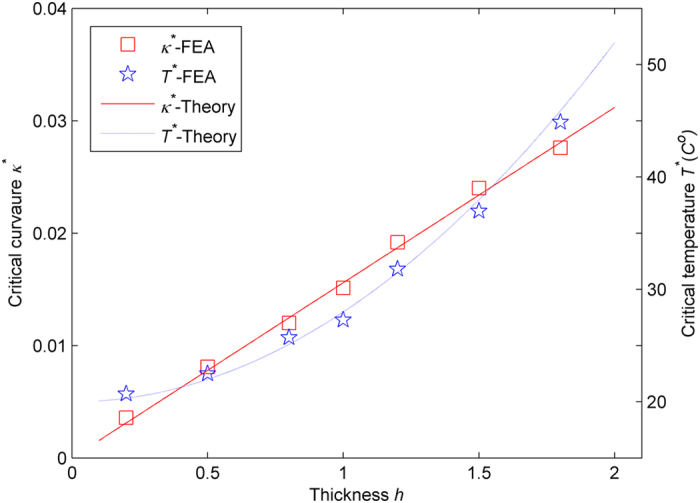
The critical curvatures and critical temperatures versus the thickness. The red squares and solid line represent the critical curvatures obtained from the numerical simulation and the theoretical analysis, respectively. The blue stars and dashed line denote the critical temperatures obtained from the simulation and the theoretical analysis, respectively.

To further verify the critical curvature κ^*^ in Eq. (9) and the critical temperature T^*^ in Eq. (10), several representative thicknesses (*h* = 0.2, 0.5, 0.8, 1, 1.2, 1.5 and 1.8) of the patterned sheet are tested computationally. The critical curvatures (blue squares) and critical temperature (blue stars) of these samples are plotted in Fig. 4. As demonstrated, their differences compared to the theoretical analysis (red solid line and blue dashed line) are fairly small.

### Numerical Simulation

To validate the critical thickness obtained from the theoretical analysis and gain more insights into the deformation of the patterned sheet, the kirigami approach to forming a buckliball from a planar patterned sheet with a thickness of *h* = 2 > h^*^ and consequent buckling-induced retraction is simulated by finite element analysis. All of the examples in this and the above sections are subject to the same experimental conditions.

In the family of stimulus-responsive hydrogels, the temperature-sensitive hydrogel poly(N-isopropylacrylamide), abbreviated as PNIPAM, is selected as the active material in the top layer because it is thermally hypersensitive and can work at normal temperature^32^. The Young’s modulus and thermal expansion coefficient of PNIPAM are 0.8 MPa and −0.005/°C, respectively^5^. The passive material in the bottom layer is a polymer, which has a Young’s modulus of 3 MPa. Its thermal expansion coefficient is negligible compared with that of the active material in the top layer. The operational temperature is set from 20 °C to 55 °C^5^, and the temperature increases linearly.

Abaqus is used for the nonlinear finite element analysis, in which the model is discretized into the C3D8R element (an 8-node linear brick with reduced integration). To eliminate friction, the top and bottom faces of the sheet are assumed to be very smooth. However, the lateral faces have to be rough; otherwise, they could peel off after contact during the bending procedure. Gravity is taken into account because it can hinder the bending and subsequently influence the final configuration^33^. To avoid rigid body motion and unsymmetrical deformation, the center point of the patterned sheet is pinned. A nonlinear finite element simulation takes requires approximately 2–3 hours for both the self-folding and retraction processes.

**Figure 5:**
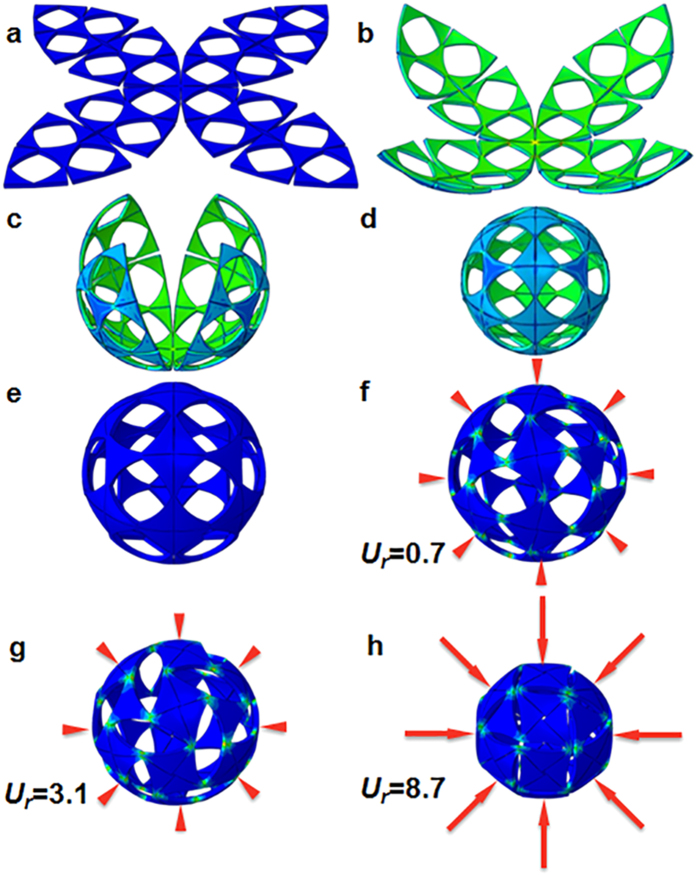
The folding procedure of a planar sheet (*h* = 2) at (**a**) 20.0 °C, (**b**) 32.6 °C, (**c**) 41.0 °C and (**d**) 45.2 °C. The buckling of this folded sheet under radially inward displacement (**e**) *u*_*r*_ = 0; (**f**) *u*_*r*_ = 0.7; (**g**) *u*_*r*_ = 3.1; and (**h**) *u*_*r*_ = 8.7. *u*_*r*_ is uniformly distributed against the external surface, and its magnitudes are represented by the lengths of the red arrows. The color indicates stress magnitude.

The initial state of the sheet at 20 °C is depicted in Fig. 5a. With increasing temperature, the active layer (top) shrinks while the passive polymer layer (bottom) retains its volume. Such a responsive disparity makes the sheet bend upward (Fig. 5b–d). In the beginning, each group (Fig. 1c) of these three base cells bends toward its center and forms a large spherical surface, as shown in Fig. 5b. Then, the bending mainly occurs at the weak junctions (e.g., Fig. 1e) and presents four curved petals as shown in Fig. 5c. Finally, the vertices of the 4 petals merge precisely, and a buckliball (Fig. 5d) is obtained at 45.2 °C.

In the self-folding process, some apertures are deformed in the horizontal direction, while the others are compressed in the vertical direction. However, the structure still exhibits a rotational symmetry, as it represents the mode with the minimal strain energy, namely, the one with the lowest eigenvalue. However, such symmetry in other potential modes cannot be held. In the mode corresponding to the second lowest eigenvalue, the buckliball deforms into an ellipsoid whose equator swells outwards. In the modes with the third and fourth eigenvalues, the buckling takes place only in specific locations, such as the two poles of the buckliball, leading to severely deformed apertures.

Because the critical temperature is as high as T^*^ = 52 °C for the thickness of *h* = 2, the entire deformation process is confined to Stage 1 and Stage 2. Thus, the unexpected buckling-induced saddle-shape and curled edges are avoided. The average external radius of this buckliball is 33.28, only 0.86% greater than the expectation (Fig. 1a). Thus, we can claim that the kirigami approach successfully meets the fabrication expectation.

To illustrate the retraction of the buckliball, the radially inward displacement *u*_*r*_ is applied uniformly on its external surface. Its maximum value has been calculated to avoid severe distortions. By increasing the displacement linearly while maintaining the temperature at 45.2 °C, the narrow ligaments of the buckliball begin to buckle (Fig. 5f). With the material rotation at the buckling points, the initial square-like apertures deform into diamond shapes (Fig. 5g), and its size becomes smaller progressively until completely squeezed (Fig. 5h). To avoid sophisticated structural interactions, the simulation is terminated when the aperture boundaries begin to contact. In the final state, the buckliball becomes a nearly enclosed ball in which the volume retraction ratio reaches 57.34%, which is very close to the theoretical limit of 59.23%. Notably, the peak stress appears in the junction region of the buckliball, as shown in Fig. 5. The self-folding process and the retraction of the buckliball are recorded in Video 1 and Video 2, respectively, which are published in the *SI*.

The processes of self-folding and volume retraction are reversible. If the radially inward displacement *u*_*r*_ varies from 8.7 to 0 linearly, the buckliball will expand into the state illustrated in Fig. 5e. Then, with the decrease of temperature, the enclosed buckliball will gradually open up and finally become flat, as shown in Fig. 5a.

Notably, the critical temperature is highly dependent on the shape of the perforated holes. By changing the shape of the internal holes in the pattern, as shown in Fig. 5a, we investigate the self-folding for a sheet (1) with circular holes and (2) without perforation. More details are given in the *SI*.

## Discussion

We propose a kirigami approach to creating highly sophisticated 3D structures via the self-folding of a bilayer patterned planar sheet. The approach is inspired by the shape transformation of some biological systems and the well-understood shape control of soft materials.

By partitioning and flattening a buckliball, we can devise a planar pattern for a sheet composed of two layers of temperature-sensitive hydrogel and a temperature-insensitive polymer. By minimizing the potential strain energy of the system, we find that the longitudinal and transverse principal curvatures respond to the increase of temperature through the following three stages: (1) uniform and linear deformation; (2) identical but nonlinear deformation; and (3) bifurcated and inversely proportional deformation. It is concluded that the spherical self-folding process can be achieved as long as the sheet thickness is above the critical value, thereby limiting the deformation to the first two stages.

The theoretical analysis is verified by the finite element simulation. The simulation indicates that the self-folding mode is highly dependent on the sheet thickness. It is also shown that the self-folding process can be altered by changing the sheet pattern. In addition, the buckling-induced retraction of the buckliball derived from the kirigami approach is simulated. Importantly, the methodology demonstrated in this study signifies a way to utilize the buckling of a structure.

Although a buckliball is used as a representative example in this paper, the kirigami approach can be used to design and fabricate more complex structures if they can be partitioned and disassembled in the same manner as the buckliball. Hierarchical patterns could be considered for the formation of complex structures and will be studied in the future. The minimal strain energy method used to determine the critical thickness and critical temperature is applicable to any arbitrary pattern as long as the surface integration in Eq. (3) can be obtained. The self-folding of a planar sheet under stimuli other than temperature can be explored using the same or similar methodology in the future for other applications. Further studies may explore applications in the areas of biological morphogenesis, micro-robotics and biomedical devices.

## Additional Information

**How to cite this article**: Lin, S. *et al*. A Kirigami Approach to Forming a Synthetic Buckliball. *Sci. Rep.* **6,** 33016; doi: 10.1038/srep33016 (2016).

## Supplementary Material

Supplementary Information

Supplementary Video 1

Supplementary Video 2
