# Supplementary material for: A Kirigami Approach to Forming a Synthetic Buckliball
Source: Sci Rep. 2016 Sep 9;6:33016. doi: 10.1038/srep33016 (PMC5017128; doi:10.1038/srep33016)
Supplement: Supplementary Information [file srep33016-s1.pdf]

A Kirigami Approach to Forming a Synthetic Buckliball

Sen Lin, Yi Min Xie, Qing Li, Xiaodong Huang, Shiwei Zhou

Supplementary information

Derivation of the strain energy density

The strain energy density U_{di} in Eq. (3) is given by:

$$U_{di}\left(E_i,\alpha_i,T\right)=\frac{1}{2}\sum\sigma_{\alpha\beta}\varepsilon^{m}_{\alpha\beta} \tag{S1}$$

For a plane stress problem, some components of the stress σ are zero e.g. $\sigma_{33} = \sigma_{23} = \sigma_{13} = 0$, then the stress-strain relation is simplified as:

$$\begin{bmatrix}\sigma_{11}\\ \sigma_{22}\\ \sigma_{12}\end{bmatrix}=\frac{E}{\left(1-\mu^2\right)}\begin{bmatrix}1 & \mu & 0\\ \mu & 1 & 0\\ 0 & 0 & \left(1-\mu\right)/2\end{bmatrix}\begin{bmatrix}\varepsilon_{11}\\ \varepsilon_{22}\\ 2\varepsilon_{12}\end{bmatrix}-\frac{E\alpha T}{\left(1-\mu\right)}\begin{bmatrix}1\\ 1\\ 0\end{bmatrix} \tag{S2}$$

where μ denotes the Poisson's ratio, α the coefficient of expansion, E the Young's modulus. Temperature T is assumed to be invariant over the sheet though it can be a space-dependent value as $T(x, y, z)$. The strain due to mechanical loading, known as elastic strain $\varepsilon^{m}{}_{\alpha\beta}$ (α,β could be 1 or 2), is given by:

$$\begin{bmatrix}\varepsilon^{m}_{11}\\ \varepsilon^{m}_{22}\\ 2\varepsilon^{m}_{12}\end{bmatrix}=\frac{1}{E}\begin{bmatrix}1 & -\mu & 0\\ -\mu & 1 & 0\\ 0 & 0 & 2\left(1+\mu\right)\end{bmatrix}\begin{bmatrix}\sigma_{11}\\ \sigma_{22}\\ \sigma_{12}\end{bmatrix} \tag{S3}$$

According to Eqs. (S1)-(S3), the strain energy density U_{di} in Eq. (3) can be obtained as

$$U_{di}=\begin{cases}(E_i(Cxy-xy(C+(\kappa_x\kappa_y)/2)+(\kappa_x\kappa_y xy)/2)^2)/(\mu+1)-(((E_i(T\alpha_i+\mu(T\alpha_i\\-y^2(C+(\kappa_x\kappa_y)/2)+Cy^2+(\kappa_x\kappa_y y^2)/2)))/(\mu^2-1)-(E_iT\alpha_i)/(\mu-1))(\mu((E_i(T\alpha_i\\-y^2(C+(\kappa_x\kappa_y)/2)+Cy^2+(\kappa_x\kappa_y y^2)/2+\mu T\alpha_i))/(\mu^2-1)-(E_iT\alpha_i)/(\mu-1))\\-(E_i(T\alpha_i+\mu(T\alpha_i-y^2(C+(\kappa_x\kappa_y)/2)+Cy^2+(\kappa_x\kappa_y y^2)/2)))/(\mu^2-1)\\+(E_iT\alpha_i)/(\mu-1)))/(2E_i)-(((E_i(T\alpha_i-y^2(C+(\kappa_x\kappa_y)/2)+Cy^2+(\kappa_x\kappa_y y^2)/2\\+\mu T\alpha_i))/(\mu^2-1)-(E_iT\alpha_i)/(\mu-1))(\mu((E_i(T\alpha_i+\mu(T\alpha_i-y^2(C+(\kappa_x\kappa_y)/2)+Cy^2\\+(\kappa_x\kappa_y y^2)/2)))/(\mu^2-1)-(E_iT\alpha_i)/(\mu-1))-(E_i(T\alpha_i-y^2(C+(\kappa_x\kappa_y)/2)+Cy^2\\+(\kappa_x\kappa_y y^2)/2+\mu T\alpha_i))/(\mu^2-1)+(E_iT\alpha_i)/(\mu-1)))/(2E_i)\end{cases}\tag{S4}$$

where C is a constant.

The geometric parameters $r_i(\theta)$ in Eq. (3) need to be clarified. If the origin of polar coordinate system is located at the center of the base cell, the inner boundaries of the base cell can be divided into 4 elliptic arcs (marked as purple curves on the left of Fig. S1). In the cylindrical coordinate system, it is defined as:

$$r_1(\theta)=\begin{cases}\sqrt{\dfrac{1}{0.00444\sin^2\theta+0.0123\cos^2\theta}} & \theta\in(-0.149,0.784)\cup(2.358,3.925)\cup(5.499,6.134)\\ \sqrt{\dfrac{1}{0.00444\cos^2\theta+0.0123\sin^2\theta}} & \theta\in(0.784,2.358)\cup(3.925,5.499)\end{cases}\tag{S5}$$

Similarly, the external boundaries r_2 (consisted of 8 straight lines) can be expressed as:

$$r_2(\theta)=\begin{cases}1/(0.0670\cos\theta+0.0526\sin\theta) & \theta\in(-0.149,0.784)\\ 1/(0.0611\cos\theta+0.0585\sin\theta) & \theta\in(0.784,1.571)\\ 1/(-0.0611\cos\theta+0.0585\sin\theta) & \theta\in(1.571,2.358)\\ 1/(-0.0670\cos\theta+0.0526\sin\theta) & \theta\in(2.358,3.291)\\ 1/(-0.0489\cos\theta-0.0678\sin\theta) & \theta\in(3.291,3.925)\\ 1/(-0.0427\cos\theta-0.0740\sin\theta) & \theta\in(3.925,4.712)\\ 1/(0.0427\cos\theta-0.0740\sin\theta) & \theta\in(4.712,5.499)\\ 1/(0.0489\cos\theta-0.0678\sin\theta) & \theta\in(5.499,6.134)\end{cases}\tag{S6}$$

However, to calculate the strain energy density over the sheet, it is necessary to consider the coordinate transformation (translation and rotation). For instance, if the origin of the local coordinate system moves to an arbitrary point (e.g. P_4 in Fig. S1), the relationship between the global coordinate (r, θ) with the origin at the lowest point of an upright petal and the local

coordinate (r', θ') originated at point P_i (r_{pi}, θ_{pi}) (i = 1, ..., 6 denotes the sequence of the base cells in the petal) is given as:

$$r = \sqrt{r'^2 + r_{pi}^2 + 2r' r_{pi} \cos(\theta' - \theta_{pi})} \quad \text{(S7)}$$

$$\theta = \arctan([r' \sin(\theta') + r_{pi} \sin(\theta_{pi})]/[r' \cos(\theta') + r_{pi} \cos(\theta_{pi})]) - \alpha_i \quad \text{(S8)}$$

where α_i is the rotation angle of the base cell with respect to the global coordinate. Because the petal shown in Fig. S1 consists of six identical base cells, the strain energy density over it can be calculated by considering the coordinate translation and coordinate rotation in Eqs. (S7)-(S8). The centers of six base cells and their rotation angle are given in Table 1 as:

Table 1: The coordinate and rotation angle of the six base cells in an upright petal.

	$i = 1$	$i = 2$	$i = 3$	$i = 4$	$i = 5$	$i = 6$
r_{pi}	84.42	65.20	65.20	39.16	39.16	17.08
θ_{pi}	4.712	4.893	4.531	4.409	5.016	4.712
α_i	0	2.094	4.189	1.047	5.236	3.142

Note that the unit of angle is radian. The strain energy density for other petals is similar to the upright petal due to its square symmetry.

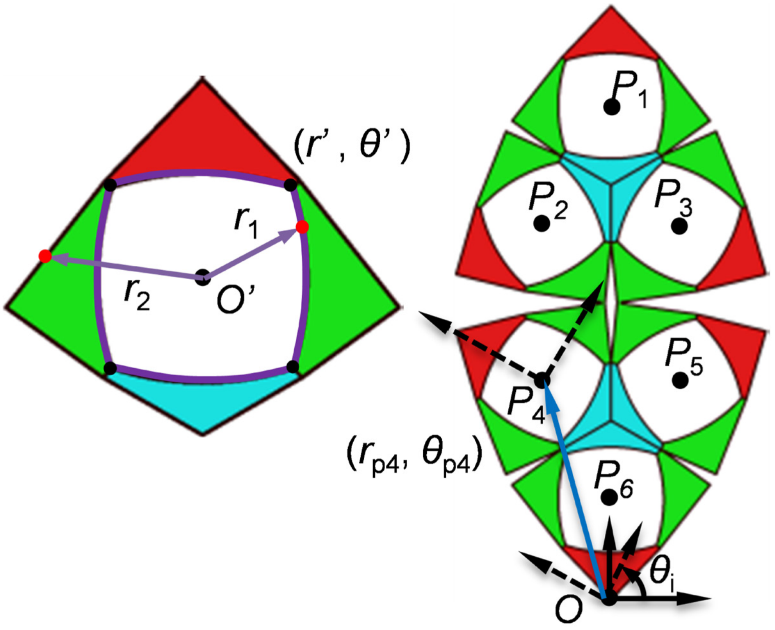

Figure. S1. The global and local coordinate systems in an upright petal.

Then the geometric parameters $r_i(\theta)$ in Eq. (3) is clarified in Eqs. (S5)-(S8) and the strain energy density per thickness U_{ti} is obtained by Eq. (3). By integrating U_{ti} across the thickness, the total strain energy U in Eq. (5) can be obtained. It is very lengthy to explicitly express U and its derivatives $\partial U/\partial \kappa_x = 0$ and $\partial U/\partial \kappa_y = 0$. Therefore we provide the following program by using the symbol calculation in MATLAB:

MATLAB Program:

```
clc;clear;
syms x1 x2 x3 A1 A2 A3 B1 B2 B3 k1 k2 E1 E2 v h h1 th r R alfa1 alfa2 T0 T
Ut11 Ut12 Ut13 Ut14 Ut15 Ut16 Ut17 Ut18 Ut1 Ut2 r1 th1 xt1 xt2 the a b
% E1 = 3000000;
% E2 = 800000;
% v = 0.3;
% h1 = 0.75*h;
% alfa1=0;
% alfa2=-0.005;
the = [0,2.094,4.189,1.047,3.142,5.236];
rp = [84.42,65.20,65.20,39.16,39.16,17.08];
thp = [4.712,4.893,4.531,4.409,5.016,4.712];

for i = 1:6
    u1 = A1*x1 + A2*x1^3 + A3*x1*x2^2;
    u2 = B1*x2 + B2*x2^3 + B3*x2*x1^2;
    u3 = k1*x1^2/2 + k2*x2^2/2;
    %%% the total strain in the plate
    e11 = 1/2*(2*diff(u1,x1) + diff(u3,x1)^2)  + k1*x3;
    e22 = 1/2*(2*diff(u2,x2) + diff(u3,x2)^2)  + k2*x3;
    e12 = 1/2*(diff(u1,x2) + diff(u2,x1) + diff(u3,x1)*diff(u3,x2));
    %%% the stress components
    s11 = E1/(1-v^2)*(e11 + v*e22)  - E1*alfa1*T/(1-v);
    s22 = E1/(1-v^2)*(e22 + v*e11)  - E1*alfa1*T/(1-v);
    s12 = E1/(1+v)*e12;
    %%% strain due to mechanical loading
    E11 = (s11-v*s22)/E1;
    E22 = (-v*s11+s22)/E1;
    E12 = (1+v)*s12/E1;
    %%% the strain energy density
    U = 1/2*[E11 E22 2*E12]*[s11 s22 s12].';
    %%% change the coordinate system
    U = subs(U,x1,r*cos(th));
    U = subs(U,x2,r*sin(th));
    U = subs(U,r,sqrt(r1^2+rp(i)^2+2*r1*rp(i)*cos(th1-thp(i))));
    U =
subs(U,th,atan((r1*sin(th1)+rp(i)*sin(thp(i)))/(r1*cos(th1)+rp(i)*cos(thp(i
))))-the(i));
    %% the total strain energy
    Ut11 =
vpa(int(int(U*r,r,(1/(0.00444*(sin(th1))^2+0.0123*(cos(th1))^2))^0.5,1/(0.0
670*cos(th1)+0.0526*sin(th1))),th1,-0.149,0.784),3);
    Ut12 =
vpa(int(int(U*r,r,(1/(0.00444*(cos(th1))^2+0.0123*(sin(th1))^2))^0.5,1/(0.0
611*cos(th1)+0.0585*sin(th1))),th1,0.784,1.571),3);
```

```
    Ut13 =
vpa(int(int(U*r,r,(1/(0.00444*(cos(th1))^2+0.0123*(sin(th1))^2))^0.5,1/(-
0.0611*cos(th1)+0.0585*sin(th1))),th1,1.571,2.358),3);
    Ut14 =
vpa(int(int(U*r,r,(1/(0.00444*(sin(th1))^2+0.0123*(cos(th1))^2))^0.5,1/(-
0.0670*cos(th1)+0.0526*sin(th1))),th1,2.358,3.291),3);
    Ut15 =
vpa(int(int(U*r,r,(1/(0.00444*(sin(th1))^2+0.0123*(cos(th1))^2))^0.5,1/(-
0.0489*cos(th1)-0.0678*sin(th1))),th1,3.291,3.925),3);
    Ut16 =
vpa(int(int(U*r,r,(1/(0.00444*(cos(th1))^2+0.0123*(sin(th1))^2))^0.5,1/(-
0.0427*cos(th1)-0.0740*sin(th1))),th1,3.925,4.712),3);
    Ut17 =
vpa(int(int(U*r,r,(1/(0.00444*(cos(th1))^2+0.0123*(sin(th1))^2))^0.5,1/(0.0
427*cos(th1)-0.0740*sin(th1))),th1,4.712,5.499),3);
    Ut18 =
vpa(int(int(U*r,r,(1/(0.00444*(sin(th1))^2+0.0123*(cos(th1))^2))^0.5,1/(0.0
489*cos(th1)-0.0678*sin(th1))),th1,5.499,6.134),3);
    Ut1 = Ut11+Ut12+Ut13+Ut14+Ut15+Ut16+Ut17+Ut18;
    Ut2 = subs(Ut1,E1,E2);
    Ut2 = subs(Ut2,alfa1,alfa2);
    phi(i) = vpa((int(Ut1,x3,0,0.75*h)+int(Ut2,x3,0.75*h,h)),3);
end

phisum = sum(phi);
%%% solve D(phi)/Ai=0 and D(phi)/Bi=0 then Substitude back into phi
S = solve(diff(phisum,A1),diff(phisum,A2), diff(phisum,A3), diff(phisum,B1),
diff(phisum,B2), diff(phisum,B3),A1,A2,A3,B1,B2,B3);

phisum = subs(phisum,A1,S.A1);
phisum = subs(phisum,A2,S.A2);
phisum = subs(phisum,A3,S.A3);
phisum = subs(phisum,B1,S.B1);
phisum = subs(phisum,B2,S.B2);
phisum = subs(phisum,B3,S.B3);
%%% D(phi)/ki=0
K1 = vpa(diff(phisum,k1),3);
K2 = vpa(diff(phisum,k2),3);
%%% Eliminating the temperature
vpa(simplify(K1-K2),3)
% solve(K1,K2,k1,k2);
```

The influence of the kirigami pattern

To further investigate the influence of the pattern on the self-folding process, the petals (1) with circular apertures and (2) without apertures are investigated. To maintain the same volume, the radius of circle is R = 10 for the first case. With the similar coordinate translation and rotation, the total strain energy U becomes

$$U = \int_0^{h_1}\int_R^{r_2}\int_0^{2\pi} U_{d1}(E_1,\alpha_1) r d\theta dr dz + \int_{h_1}^{h}\int_R^{r_2}\int_0^{2\pi} U_{d2}(E_2,\alpha_2) r d\theta dr dz \tag{S9}$$

By letting $\partial U/\partial \kappa_x = 0$ and $\partial U/\partial \kappa_y = 0$, we obtain:

$$7934\kappa_x\kappa_y^2 + 2.72h^2\kappa_x + 0.81h^2\kappa_y - 0.0109Th = 0 \tag{S10}$$

$$7934\kappa_x^2\kappa_y + 2.72h^2\kappa_y + 0.81h^2\kappa_x - 0.0109Th = 0 \tag{S11}$$

Eliminating the temperature T in Eqs. (S10) and (S11) leads to:

$$(\kappa_x - \kappa_y)(4153\kappa_x\kappa_y - h^2) = 0 \tag{S12}$$

Similarly the critical curvature can be calculated as:

$$\kappa^* = \kappa_x = \kappa_y = h/64.44 \tag{S13}$$

By substituted Eq. (S13) into Eq. (S10)**,** the curvature components are eliminated to yield:

$$T^* = 7.75h^2 \tag{S14}$$

Eqs. (S13)-(S14) show the notable difference of the critical curvature and critical temperature when the aperture shape changes from a cornered square to a circle with the same area. Note that the influence of pattern is more remarkable in Stage 3. The disparity of longitudinal principal curvature and transverse principal curvature for the sheet with the circular aperture is much greater than that with the cornered square. The reason could be attributed to the thinner ligament (junctions) form in the latter at which more energy can be absorbed, making the main parts undergo relatively less deformation and, therefore, less disparity in the principal curvatures.

In order to obtain an explicit expression for the dependence of curvature on temperature, we conduct the following dimensionless analysis. For $T < 7.75h^2$ ($\kappa_x = \kappa_y$), the dimensionless curvature is defined as $\bar{\kappa}_1 = 64.44\kappa_x/h = 64.44\kappa_y/h$ and it allows $0 < \bar{\kappa}_1 < 1$. While a dimensionless temperature can be similarly defined as $\bar{T} = T/(7.75h^2)$. Substituted $\bar{\kappa}_1$ into Eq. (S10), the relationship between $\bar{\kappa}_1$ and $\bar{T}$ is obtained as:

$$\overline{T} = 0.351\overline{\kappa_1}^3 + 0.649\overline{\kappa_1} \tag{S15}$$

or

$$\overline{\kappa_1} = \left(1.42\overline{T} + \left(2.03\overline{T}^2 + 0.234\right)^{\frac{1}{2}}\right)^{\frac{1}{3}} - \frac{0.616}{\left(1.42\overline{T} + \left(2.03\overline{T}^2 + 0.234\right)^{\frac{1}{2}}\right)^{\frac{1}{3}}} \tag{S16}$$

For $T > 7.75h^2$ where the two principal curvatures begin to deviate, the dimensionless curvature is adopted as $\overline{\kappa_2} = h/64.44\kappa_x = 64.44\kappa_y/h$. Substituted $\overline{\kappa_2}$ into Eq. (S10), the relationship between $\overline{\kappa_2}$ and $\overline{T}$ is found as:

$$\overline{T} = 0.5\left(\overline{\kappa_2} + 1/\overline{\kappa_2}\right) \quad \text{or} \quad \begin{cases} \overline{\kappa_{2a}} = \overline{T} + \left(\overline{T}^2 - 1\right)^{\frac{1}{2}} \\ \overline{\kappa_{2b}} = \overline{T} - \left(\overline{T}^2 - 1\right)^{\frac{1}{2}} \end{cases} \tag{S17}$$

When thickness $h = 0.2$, Eq. (S16) can be plotted as the dashed black line in Fig. S2. Its difference from the numerical results (red dots) is very small. Thus, the theoretical analysis is further verified.

For the case of the pattern without apertures (which corresponding to $R = 0$ for the circular aperture), all the parameters are invariant except for the internal boundaries $r_1 = 0$. Therefore, the strain energy density per thickness becomes

$$U = \int_0^{h_1}\int_0^{r_2}\int_0^{2\pi} U_{d1}(E_1, \alpha_1) r d\theta dr dz + \int_{h_1}^{h}\int_0^{r_2}\int_0^{2\pi} U_{d2}(E_2, \alpha_2) r d\theta dr dz \tag{S18}$$

Then those two equations about κ_i can be similarly obtained as,

$$11802\kappa_x\kappa_y^2 + 5.73h^2\kappa_x + 1.91h^2\kappa_y - 0.0251Th = 0 \tag{S19}$$

$$11802\kappa_x^2\kappa_y + 5.73h^2\kappa_y + 1.91h^2\kappa_x - 0.0251Th = 0 \tag{S20}$$

Eliminating the temperature from above equations leads to:

$$\left(\kappa_x - \kappa_y\right)\left(3089\kappa_x\kappa_y - h^2\right) = 0 \tag{S21}$$

The critical curvature can be calculated as:

$$\kappa^* = \kappa_x = \kappa_y = h / 55.58 \tag{S22}$$

Similarly to the previous step, eliminating the curvature to obtain the critical temperature as:

$$T^* = 8.22h^2 \tag{S23}$$

Compared with Eq. (S14), the critical temperature for the hole-free pattern considerably increases if the sheet thickness remains the same. It agrees with the common knowledge that more material requires higher energy to reach the critical point.

Under the same critical temperature 45.2°C, the sheet with the hole-free pattern has a critical thickness of $h^* = 1.75$ to generate a perfect spherical configuration, which is slightly smaller than the one ($h^* = 1.78$) in the manuscript.

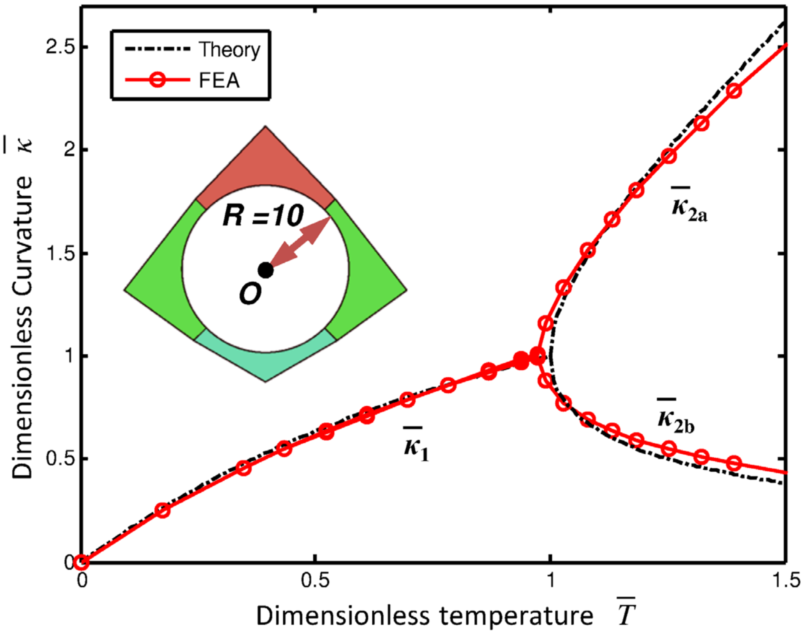

Figure. S2. The relationship between the dimensionless principal curvatures and dimensionless temperature for the petals with a circular pattern.

For $T < 8.22h^2$, the structure bends in two equal principal curvatures. The dimensionless curvature is adopted as $\overline{\kappa}_1 = 55.58\kappa_x/h = 55.58\kappa_y/h$ ($0 < \overline{\kappa}_1 < 1$), while a dimensionless temperature can be similarly defined as $\overline{T} = T/(8.22h^2)$. Substituting $\overline{\kappa}_1$ into Eq. (S19) establishes the relationship between $\overline{\kappa}_1$ and $\overline{T}$ as,

$$\overline{T} = 0.333\overline{\kappa_1}^3 + 0.667\overline{\kappa_1} \tag{S24}$$

or

$$\overline{\kappa_1} = \left(1.50\overline{T} + \left(2.25\overline{T}^2 + 0.298\right)^{\frac{1}{2}}\right)^{\frac{1}{3}} - \frac{0.668}{\left(1.50\overline{T} + \left(2.25\overline{T}^2 + 0.298\right)^{\frac{1}{2}}\right)^{\frac{1}{3}}} \tag{S25}$$

For $T > 8.22h^2$, the structure has two unequal principal curvatures as $\overline{\kappa}_2 = h/55.58\kappa_x = 55.58\kappa_y/h$.

Similarly, when thickness $h = 0.2$, Eq. (S24) is plotted and verified with the numerical simulation in Fig. S3. In this case, the theoretical prediction (dashed black line) and numerical results (red marks) match very well in Stage 1 and Stage 2. However, the disparity of theoretical principal curvatures is slightly smaller than the numerical counterpart. The reason can be explained by the artificially broadened junctions in Fig. 1e, which are not considered in the theoretical analysis but they indeed weaken the degree of self-folding in the numerical simulation.

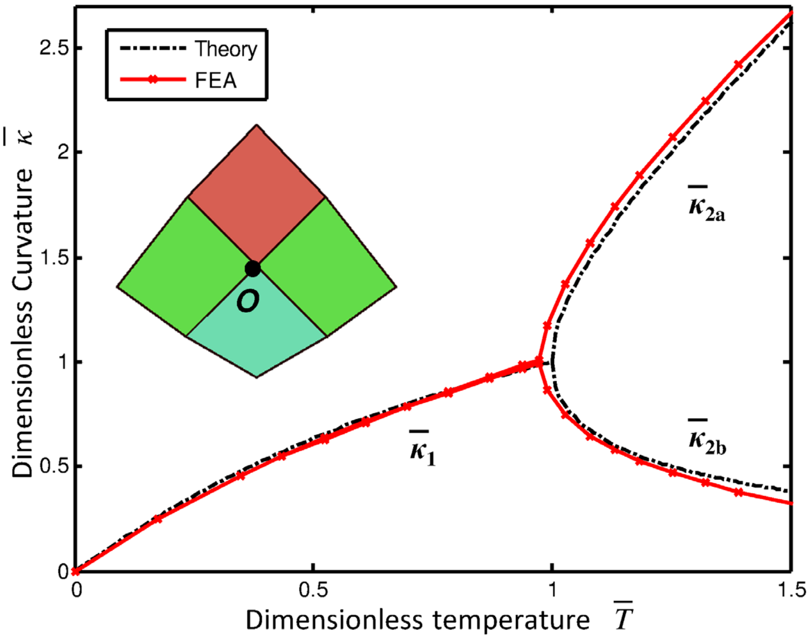

Figure. S3. The relationship between the dimensionless principal curvatures and dimensionless temperature for the petals with the hole-free pattern.

Derivation of mid-plane displacements

The mid-plane strain ε_x, ε_y and mid-plane shear stain γ_{xy} can be defined by the following equations:

$$\begin{cases} \varepsilon_x = \dfrac{\partial u_1}{\partial x} + \dfrac{1}{2}\left(\dfrac{\partial u_3}{\partial x}\right)^2 \\ \varepsilon_y = \dfrac{\partial u_2}{\partial y} + \dfrac{1}{2}\left(\dfrac{\partial u_3}{\partial y}\right)^2 \\ \gamma_{xy} = \dfrac{\partial u_1}{\partial y} + \dfrac{\partial u_2}{\partial x} + \left(\dfrac{\partial u_3}{\partial x}\right)\left(\dfrac{\partial u_3}{\partial y}\right) \end{cases} \tag{S26}$$

In Eq. (1), u_3 is assumed as $u_3 = \kappa_x x^2/2 + \kappa_y y^2/2$, which indicates four possibilities: (1) a spherical shape with $\kappa_x = \kappa_y$; (2) a saddle shape with $\kappa_x\kappa_y < 0$; (3) an ellipsoidal shape with $\kappa_x < \kappa_y$ and $\kappa_x\kappa_y > 0$; and (4) an ellipsoidal shape with $\kappa_x > \kappa_y$ and $\kappa_x\kappa_y > 0$. With the assumption that ε_x is a function of y, ε_y is a function of x and the mid-plane shear stain $\gamma_{xy} = 0$, the in-plane deformations u_1 and u_2 in Eq. (1) can be obtained as:

$$
\begin{cases}
u_1(x,y) = C_1 x - \dfrac{\kappa_x^2 x^3}{6} - \dfrac{\kappa_x \kappa_y x y^2}{4} \\
u_2(x,y) = C_2 y - \dfrac{\kappa_y^2 y^3}{6} - \dfrac{\kappa_x \kappa_y x^2 y}{4}
\end{cases}
\tag{S27}
$$

where C_1 and C_2 are the constants.

Video 1. The folding procedure of a planar sheet

Video 2. The buckling of a folded buckliball under radial-inward displacement
