# Supplementary figures and images for: A Kirigami Approach to Forming a Synthetic Buckliball
Source: Sci Rep. 2016 Sep 9;6:33016. doi: 10.1038/srep33016 (PMC5017128; doi:10.1038/srep33016)

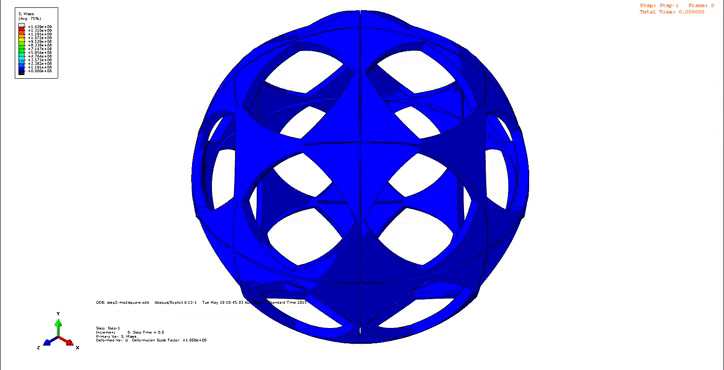

Supplement: Supplementary Video 1 [file srep33016-s2.gif]

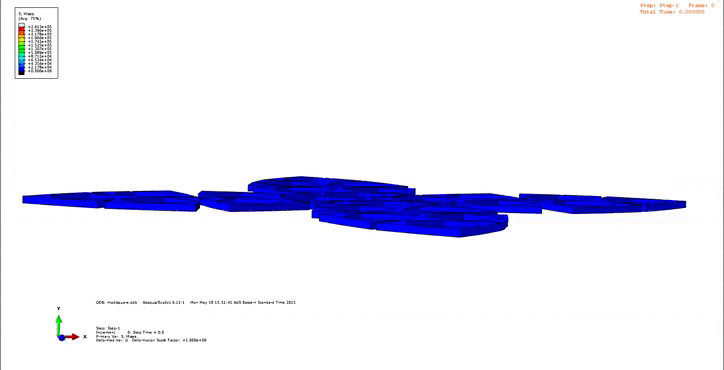

Supplement: Supplementary Video 2 [file srep33016-s3.gif]
